# A Methodology for Simply Evaluating the Safety of a Passenger Ship Stability Using the Index for the Intact Stability Appraisal Module

**DOI:** 10.3390/s22051938

**Published:** 2022-03-02

**Authors:** Donghan Woo, Nam-Kyun Im

**Affiliations:** Department of Navigation Science, Mokpo National Maritime University, Mokpo-si 58628, Jeollanam-do, Korea; woodh@mmu.ac.kr

**Keywords:** ship stability, GZ curve, IMO stability regulations, stability assessment

## Abstract

To evaluate the safety of passenger ships’ stability, ten stability parameters should be calculated. However, since the process for calculating all stability parameters is complex without a ship loading program, a convenient methodology to simply calculate them and evaluate the safety condition of a passenger ship is required to alert the hazard to a captain, officer, and crew. The Index for Passenger Ship Intact Stability Appraisal Module (IPSAM) is proposed herein. According to the value of a passenger ship’s metacentric height (GM) which could be calculated by the ship’s roll period measured by sensors in real-time, IPSAM simply calculates nine intact stability parameters except for $Angle_{maxGZ}$ and proposes the present stability status as a Single Intact Stability Index (SISI). It helps crews easily recognize the safety of passenger ships’ stability as a decision support system in real-time. Based on the intact stability parameters of 331 loading conditions of 11 passenger ships, empirical formulas for IPSAM were derived. To verify the empirical formulas of IPSAM, the stability parameters of a passenger ship in 20 loading conditions were calculated using proposed empirical formulas and the principal calculation methods respectively, then compared. Additionally, the result of the SISI of 20 loading conditions successfully indicates the danger as the value of the SISI under 1.0 of the three loading conditions that do not satisfy the IMO intact stability requirements.

## 1. Introduction

**Figure 1:**
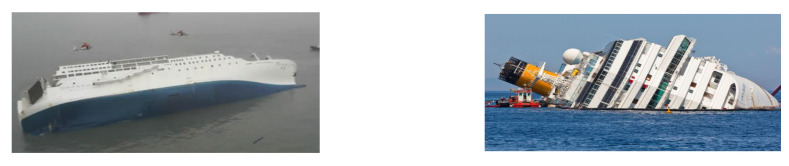
Seowol passenger ship (**left**) and Costa Concordia cruise ship (**right**) accidents [3,4].

Safety of Life At Sea (SOLAS) has been a high priority in the passenger ship industry since the accident of Titanic accident in 1912 to ensure that safety levels not only remain high but continuously increase [1]. Monitoring the safety of ships’ stability according to International Maritime Organization (IMO) is necessary to safely operate passenger ships [2]. The lack of ships’ stability can lead to capsizing incidents causing a mass mortality tragedy and immeasurable loss of property. Recent accidents, such as the Sewol passenger ship accident caused 304 fatalities in the disaster, including around 250 students on 16 April 2014 in Byeongpungdo, the Republic of Korea, and the Costa Concordia cruise ship accident caused 32 fatalities on 13 January 2012 in the Tyrrhenian sea, Italy as shown in Figure 1 [3,4].

These accidents show that despite the efforts of IMO for raising the stability requirements, there remains work for increasing the awareness of the vulnerability and safety level of ships’ stability [1]. An easily understandable decision support module for crews is necessary for accurate and timely response in case of the risk of stability.

Before establishing the standards for ships’ stability from IMO, there were several studies for laying the foundation of ship stability in the 1930s [5]. Pierrottet proposed the basic concept of the standards of stability for ships [6]. The basic concept includes the weather criterion of ships’ stability. The methodology of the assessment of the certainty of the amount of ships’ stability parameters was proposed [7]. The stability criteria for various types of ships have been developed by IMO and culminated in the completion of a Code. The International Standard (IS) Code includes the fundamentals for the safety of ships’ stability [8]. These criteria are regarded as the first-generation of the intact stability criteria [9,10]. In accordance with the SOLAS 1960 Convention, the first intact stability regulations were originated [11]. The Intergovernmental Maritime Consultative (IMCO) (Res. A 167, 168) proposed the general stability criteria based on righting arm characteristics. Evaluating naval ships’ stability using the IMO intact stability regulations was inappropriate due to the differences in the characteristics compared to general ships [12]. Mantari et al. [13] commented on the limitations of the 2008 IS code, part B in the aspect of the prevention of stability failure due to the combined effect of fishing gear pull, beam wave, and wind. Hu et al. [14] proposed the stability criterion of the sail-assisted ships and how to calculate the parameters and determine specific coefficients for the improvement of the IMO intact stability regulations. The weather criteria for the IMO intact stability regulations in the river-sea were proposed [15].

The application of existing intact stability regulations is not appropriate because of the advancement of the design technologies of large and modern ships such as container and car carriers and Ropax [16]. The second-generation intact stability criteria were established for the improvement of the IMO intact stability regulations in 2001 [11]. Umeda et al. [17] proposed the initial studies of capsizing, broaching, and parametric rolling for the improvement of the second-generation intact stability criteria. Bulian and Francescutto [18] proposed the second-generation intact stability criteria for pure loss of stability and broaching-to. The estimation methodology of the parametric rolling and pure loss in longitudinal waves was introduced [19,20,21]. Hasanudin and Chen [22] stated the limitation of the IMO intact stability regulations which was only applicable to ships navigating on a calm sea. Tompuri el al. [23] suggested the operational limitations for ships complying with the second-generation intact stability requirements, and therefore it is important to evaluate these at an early design stage. Chung et al. [24] suggested possible technical solutions for the improvement of the second-generation intact stability criteria.

There have been many studies on the convenient functionality for the assessment of intact stability from the perspective of the ships’ crew. Estimating ships’ stability using roll period in real-time was validated [25]. Terada et al. [26] proposed the methodology to calculate metacentric height (GM) using roll-period data. Santiago et al. [27] estimated the stability of fishing vessels based on the analysis of roll motion and the mass moment of inertia. The stability monitoring system estimated the GM of fishing vessels using the measured roll angle [28]. Chen et al. [29] used a machine-learning algorithm to efficiently detect the unknown parameters of a ship motion model. The elliptic Fourier descriptors from the unusual pattern recognition and classification problems were introduced for assessing wind loads on marine structures [30]. Ariffin et al. [31] used radar and a buoy for a real-time stability evaluation system. Based on the experimental data for the wave height causing fishing vessels to capsize, the estimation function was developed [32]. Daekin [33] suggested the guideline for the stability assessment for ships’ crew. Gonzales et al. [34] created an assistant system to provide information on fishing vessels’ level of stability in an easily understandable way. The relationship between key elements of the intact stability risk was proposed [35]. The system to categorize the four-level ship stability safety was proposed to help Search and Rescue (SAR) operators and ship crew [36]. Im and Choi [5] proposed the stability index calculation module for the assessment of ten IMO intact stability parameters which can help ships’ crew to easily understand the risk of ships’ stability. Toan et al. [37] developed the numerical methodology using mathematical programming to efficiently estimate the ships’ optimal hydronamic parameters in the early design stage. Emillia et al. [38] developed the system to evaluate the ships’ stability using an automatic information system based on the limited curve.

The Index for Passenger Ships’ Intact Stability Appraisal Module (IPSAM) is proposed as a decision support system to determine whether a passenger ship can comply with IMO intact stability regulations. It could be used as a supplement methodology for passenger ships’ crew to conveniently examine the level of the safety of the stability in real-time. Based on ten IMO intact stability parameters of 331 loading conditions of 11 passenger ships, the empirical formulas of IPSAM were derived to calculate stability parameters according to GM. In the stability index calculation module of IPSAM, nine stability parameters except for $Angle_{maxGZ}$ are used to develop the stability index, which has the advantage of being able to quantify the ship and presents stability status as a Single Intact Stability Index (SISI). To verify empirical formulas of IPSAM, the stability parameters of a passenger ship in 20 loading conditions were calculated by derived empirical formulas herein and the principal calculation methods respectively, then compared. Additionally, the results of the SISI of 20 loading conditions successfully evaluate the insufficient stability status of the passenger ship. The IPSAM will be able to be used as a decision support system for passenger ship’s crew to estimate the safety of the stability according to the GM calculated by roll periods measured by a sensor in real-time.

## 2. IMO Intact Stability Parameters

The ten IMO intact stability criteria are required for passenger ships to evaluate the status of their stability safety. The IMO intact stability parameters were used to derive the empirical equations in IPSAM. Table 1 shows the IMO intact stability parameters and their criteria. Eight stability parameters from No 1 to No 8 are the general criteria for cargo ships. Passenger ships are required to comply with two additional requirements as $Angle_{passenger}$ and $Angle_{turning}$.

**Figure 2:**
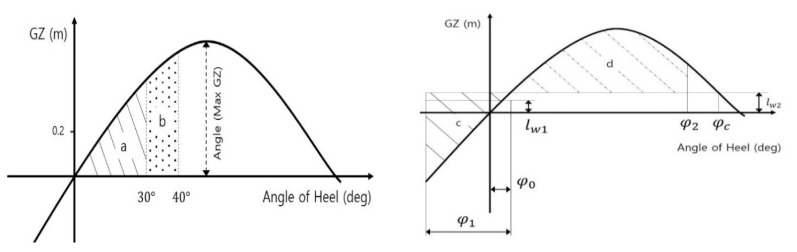
Areas under the GZ curve (**Left**) Weather criterion (**Right**) [5].

GM is the length between the center of gravity and the transverse metacenter. $GZ_{30deg}$ is the righting arm at 30° of the heeling angle. $Angle_{maxGZ}$ is the angle at the moment of the maximum GZ value in the range. Figure 2 shows three areas as “a” and “b”, except for that under the GZ curve [5]. $Area_{0-30deg}$,$Area_{30-40deg}$, and $Area_{0-40deg}$ mean the area under the GZ curve according to their mentioned angle of heeling ranges in the symbols. The steady wind pressure on the ship leads to a heeling arm ($l_{w1}$) as shown in the weather criterion of Figure 2. It leads to a heel angle ($\varphi_{0}$) for the equilibrium, and the wave motion causes the ship to heel with an angle ($\varphi_{1}$) against the wind. At this moment, the angle ($\varphi_{1}$) should be lower than the value of 16° or 80% of the immersion angle. The area of “d” should be larger than the area of “c” in the inclination of the ship due to the gust wind pressure.

The heeling angle of $Angle_{turning}$ and $Angle_{passenger}$ on account of the passenger ship’s turning and the crowding of passengers to one side respectively needs to be evaluated for passenger ships. The heeling angle ($Angle_{turning}$) generated by the rudder operation needs to be less than 10°. The heeling angle can be calculated by applying the balance between the turning moment and static righting moment using the GZ curve and the moment obtained by the following Equation (1).

(1) $$M_{R}=0.200\Delta \left(\frac{V_{0}^{2}}{LWL}\right)\left(KG-\frac{dr}{2}\right)$$

where, $M_{R}$ denotes the passenger ship’s heeling moment by turning (kNm), $V_{s}$ denotes the passenger ship’s service speed (m/s), LWL denotes the length of the ship at the waterline (m), Δ denotes the displacement (t) of the ship, dr denotes the mean draught (m), and KG denotes the height of the center of gravity above the baseline (m).

The heeling angle ($Angle_{passenger}$) on account of the crowding of passengers to one side should not exceed 10°. The Ministry of Oceans and Fisheries of the Korean Government introduced simplified empirical formulas to easily calculate the heeling angle ($Angle_{passenger}$) using the basic dimensions of a ship as shown in Equation (2) [39].

(2) $$M_{P}=\frac{0.214\left(\sum^{\;}\left(\frac{7}{m^{2}}-\frac{n}{a}\right)n\cdot b\right)}{100}$$

where, $M_{P}$ denotes the heeling moment caused by the crowding of passengers to one side of a passenger ship (kNm), n denotes the number of passengers at each passenger site, a denotes the floor area at each passenger site ($m^{2}$), b denotes the average lateral movement distance of passengers in passenger-accessible places (m).

## 3. Concept of IPSAM

**Figure 3:**
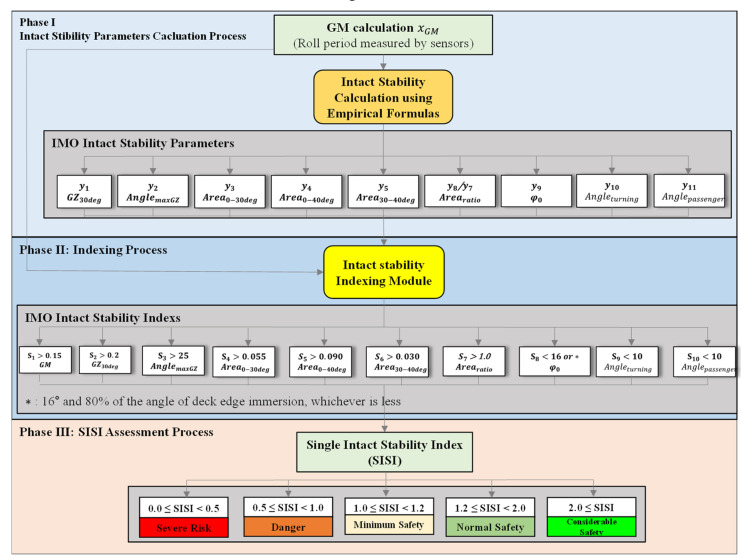
Concept of Index for Passenger Ships’ Intact Stability Appraisal Module (IPSAM).

IPSAM simply calculates nine intact stability parameters except for $Angle_{maxGZ}$ and proposes the present stability status as SISI. It helps crews easily recognize the safety of passenger ships’ stability as a decision support system. Figure 3 illustrates the detail of IPSAM of the flow chart of the three phases. In Phase Ⅰ, the GM was calculated using the simple calculation equation or roll period measured by the sensors. The eight IMO intact stability parameters were calculated using empirical formulas according to the GM in Phase Ⅰ. In Phase Ⅱ, the indexing intact stability parameters were processed. In Phase Ⅲ, all IMO stability parameter indexes were calculated to SISI, and then their value was assessed as five risk levels as shown in Figure 3.

### 3.1. Empirical Formulas of IPSAM

**Figure 4:**
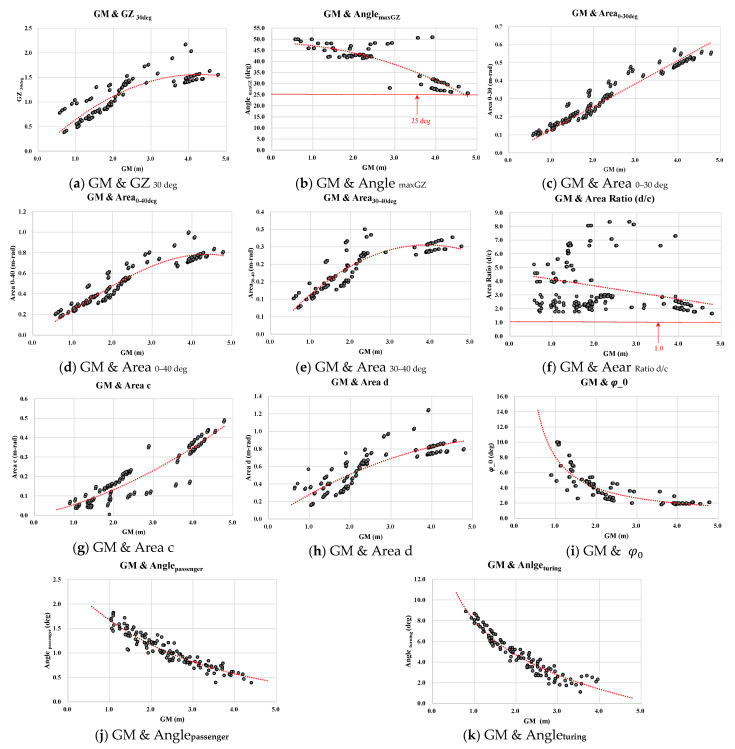
Trend lines of the seven IMO intact stability parameters according to GM.

To derive empirical formulas for calculating nine IMO intact stability parameters according to the GM, this study used 331 loading condition data of 11 passenger ships. Two types of passenger ships were introduced as Car Ferry and Ropax. These two types are dominant in the passenger ships industry. The passenger ships’ basic particulars of the 11 ships are presented in Table 2. The range of full displacement of passenger ships was from 4318.8 to 16,044.7 tons and the block coefficient was from 0.475 to 0.765 in 331 loading conditions. Empirical formulas to easily calculate IMO intact stability parameters including $Area_{c}$ and $Area_{d}$ using the GM were derived herein as shown in Table 3. Figure 4 illustrates the correlation graph of the nine stability parameters and the additional two areas of “c” and “d” of weather criterion according to GM. The method of least squares was introduced to derive empirical formulas which have the highest correlation to improve the coefficient of the determination of them as shown in Table 3.

Derived empirical formulas were analyzed for evaluating whether they could be properly used as discriminants for IMO intact stability regulations or not as follows: the nine empirical formulas except for $Angle_{maxGZ}$ and $Area_{ratio}\;$ had the high coefficient of the determination $R^{2}$ as over 0.75 as shown in Table 3. In other words, these empirical formulas reliably reflected the relationship between the four parameters and GM. Before reaching $Angle_{maxGZ}$, the GZ curve showed the simple increasing linear function with the angle of heeling as shown in Figure 2. Thus, the empirical formulas of $GZ_{30deg}$, $Area_{0-30deg}$,$\;Area_{0-40deg}$,$Area_{30-40deg}$ according to the GM showed the high coefficient of the determinations $R^{2}$ as 0.7657, 0.9525, 0.9112, and 0.8126 respectively.

However, it is problematic for $Angle_{maxGZ}$ to derive the proper formula to estimate it according to only a single variable of GM. The derivation of the correlation function of $Angle_{maxGZ}$ with a single variable of GM to calculate the angle of heel at the maximum point of the sinusoidal function of the GZ curve is unreliable. In the previous study, the method of the prediction of stability parameters according to ships’ geometrical particulars such as the breadth, depth, and draught at the concept design stage were proposed [40]. It demonstrated the high coefficient of the determination between stability parameters and ships’ geometrical particulars which are constant variables. To improve the empirical formulas of $Angle_{maxGZ}$ according to the GM calculated by the roll-period measured by a sensor in real-time while navigating needs additional further studies with the consideration of additional ships’ variables while being operated on the sea. Thus, this study does not employ the empirical equation of $Angle_{maxGZ}$ for IPSAM.

Additionally, due to the low correlation shown as (f) in Figure 4 and Table 3, using the empirical formula of $Area_{ratio}\;$ is problematic. The coefficient of the determination $R^{2}$ of the empirical formula of $Area_{ratio}$ was under 0.1 as shown in Table 3. Instead of using the empirical formulas of $Area_{ratio}$, the empirical formulas of $Area_{c}$ and $Area_{d}$ which have the high determination $R^{2}$ as 0.8754 and 0.7707 respectively were used for calculating $Area_{ratio}$. The scale of the graph of $Area_{d}$ was two times bigger than $Area_{c}$ as shown in (g) and (h) of Figure 4. Thus, it could be expected that most of the loading conditions could be evaluated as satisfying the IMO intact stability requirement of $Area_{ratio}$ as over 1.0.

### 3.2. IMO Intact Stability Parameter Index Formulas

The IPSAM introduced Equations (3)–(10) proposed by Im and Choe [5]. The ten stability parameters are normalized to the IMO stability parameters index $\left(SP_{i}\right)$ using those equations. There are two groups as follows: the first group includes the seven IMO stability parameter indexes $\left(SP_{1}-SP_{7}\right)$ which need to have higher values than their standards and the second group includes the three IMO stability parameter indexes $\left(SP_{8}-SP_{10}\right)$ which need to have lower values than their standards. The two groups use the different formulas as below.

(3) $$SP_{i}=k\left(\frac{y_{i-1}}{y_{i-1\;\left(IMO\right)}}\right)\;\;\;\;\;\;\;\;\;\;\;\;\;\;\;\;\;\;\;\;\;\;\;\;\;\;\;\;\;\;\left(0\leq y_{i-1}<y_{i-1\;\left(IMO\right)}\right)$$

(4) $$SP_{i}=k+\left(1-k\right)\left(\frac{y_{i-1}-y_{i-1\;\left(IMO\right)}}{y_{i-1\;\left(SafetyLimit\right)}-y_{i-1\;\left(IMO\right)}}\right)\;\;\;\;\;\;\;\;\;\;\;\;\;\left(\;y_{i-1\;\left(IMO\right)}\leq y_{i-1}<y_{i-1\;\left(SafetyLimit\right)}\right)$$

(5) $$SP_{i}=1+\frac{y_{i-1}-y_{i-1\;\left(SafetyLimit\right)}}{y_{i-1\;\left(FullLoading\right)}-y_{i-1\;\left(SafetyLimit\right)}}\;\;\;\;\;\;\left(\;y_{i-1\;\left(SafetyLimit\right)}\leq y_{i-1}<y_{i-1\;\left(FullLoading\right)}\right)$$

(6) $$SP_{i}=2+\frac{y_{i-1}-y_{i-1\;\left(FullLoading\right)}}{y_{i-1\;\left(FullLoading\right)}}\;\;\;\;\;\;\;\;\;\;\;\;\;\;\;\;\;\;\left(\;y_{i-1\;\left(FullLoading\right)}\leq y_{i-1}\right)$$

(7) $$SP_{i}=k\left|\frac{Angle_{coeff}-y_{i-1}}{Angle_{coeff}-y_{i-1\;\left(IMO\right)}}\right|\;\;\;\;\;\;\;\;\;\;\;\;\;\;\;\left(Angle_{coeff}\geq y_{i-1}>y_{i-1\;\left(IMO\right)}\right)$$

(8) $$SP_{i}=k+\left(1-k\right)\left|\frac{y_{i-1}-y_{i-1\;\left(IMO\right)}}{y_{i-1\;\left(SafetyLimit\right)}-y_{i-1\;\left(IMO\right)}}\right|\;\;\;\;\;\;\;\;\;\;\;\left(y_{i-1\;\left(IMO\right)}\geq y_{i-1}>y_{i-1\;\left(SafetyLimit\right)}\right)$$

(9) $$SP_{i}=1+\left|\frac{a_{i}-a_{i-SafetyLimit}}{a_{i-FullLoading}-a_{i-SafetyLimit}}\right|\;\;\;\;\;\;\;\;\;\;\;\;\;\;\;\;\left(y_{i-1\;\left(SafetyLimit\right)}\geq y_{i-1}>y_{i-1\;\left(FullLoading\right)}\right)$$

(10) $$SP_{i}=2+\left|\frac{y_{i-1}-y_{i-1\;\left(FullLoading\right)}}{y_{i-1\;\left(FullLoading\right)}}\right|\;\;\;\;\;\;\;\;\;\;\;\;\;\;\left(y_{i-1\;\left(FullLoading\right)}\geq y_{i-1}\right)$$

where, k denotes that the stability index coefficient as 0.5 to indicate 50% of parameters of the IMO requirements in Equations (3) and (4), $y_{i-1\;\left(SafetyLimit\right)}$ denotes that all stability parameters satisfy the IMO stability regulations for the first, $y_{i-1\;\left(FullLoading\right)}$ denotes that all stability parameters satisfy the IMO stability regulations in the loading condition of a ship in the fully loaded departure condition, $Angle_{coeff}$ denotes the maximum heel angle coefficient, $SP_{1}$ denotes the IMO stability parameter index for GM as $y_{0}$, $SP_{6}$ denotes the IMO stability parameter index for $Area_{ratio}$ using Area c and Area d as $y_{7}$ and $y_{8}$ respectively as shown in Table 3.

Equation (11) is used for calculating the Single Intact Stability Index (SISI). SISI is determined as the average of the stability indexes.

(11) $$SISI=\frac{\sum_{i=1}^{n}SP_{i}}{9}$$

## 4. Validation of IPSAM

### 4.1. Particular of Passenger Ship for Validation

In the present study, the passenger ship which is a type of car ferry was introduced to validate the IPSAM. The basic particulars of the passenger ship are presented in Table 4. The passenger ship was a 15,180 tons class passenger ship serviced on the coast in the Republic of Korea and it could accommodate 1500 persons. Its deadweight was 5527 tons.

### 4.2. Verification of Empirical Formulas of IPSAM

The principal methodology was used to calculate ten IMO intact stability parameters of a variety of loading conditions of the passenger ship. There were different 20 loading conditions including three unsatisfied loading conditions with the IMO intact stability criteria for the passenger ship as shown in Table 5. To verify the derived empirical formulas herein, eight IMO intact stability parameters except for $Angle_{maxGZ}$ were calculated using them, and error ratios were compared to parameters calculated by the principal methodology as shown in Table 6. The parameters marked in red were incompliant with the IMO intact stability requirements in Table 6. The results calculated by the derived empirical formulas successfully evaluated the unsatisfied parameters and whether they complied with the IMO regulations or not for loading conditions No. 1, 2, and 3 as shown in Table 6. This meant that the empirical formulas reliably reflected the relationships between the ten IMO intact stability parameters of the passenger ship and the GM.

**Figure 5:**
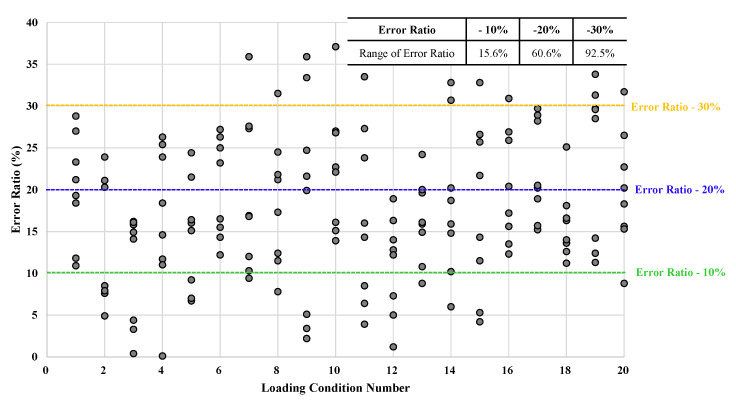
Error ratio of IMO intact stability parameters calculated by empirical formulas.

As mentioned in Section 3.1, instead of using the empirical formula of $Area_{ratio}$ which has the lowest coefficient of the determination $R^{2}$ as 0.0993, the empirical formulas of $Area_{c}$ and $Area_{d}$ which have the high determination $R^{2}$ as 0.8754 and 0.7707 respectively were used for calculating $Area_{ratio}$ herein. As shown in Table 6, the parameter of $Area_{ratio}$ using the methodology complied with the IMO intact stability requirement as 1.0 in all loading condition cases. As stated in Section 3.1, the scale of the graph of $Area_{d}$ was two times bigger than $Area_{c}$ as shown in (g) and (h) of Figure 5. Thus, $Area_{ratio}$ could be considered as generally compliant with the requirement as 1.0 in most loading conditions. The parameters which had over 20% and 30% of error ratio are marked with blue and yellow colors respectively in Table 6. The error ratios of the parameters under 30%, 20%, and 10% were 92.5%, 60.6%, and 15.6% respectively as shown in Figure 5. The average error ratio of all IMO intact stability parameters calculated by the empirical formulas was 17.9%. To improve the reliability of the empirical formulas, the average error ratio should be reduced. Thus, it was expected that the increase of the number of various passenger ships’ loading conditions would improve the reliability of the empirical formulas for estimating IMO intact stability parameters using the GM calculated by the roll period measured by the sensor. As mentioned in Section 3.2, the eight IMO intact stability parameters of the loading case No.4 in Table 6 had all stability parameters satisfy the IMO stability regulations for the first time with $y_{i-1\;\left(SafetyLimit\right)}$ for the process of the IMO intact stability indexes.

### 4.3. Verification of SISI of IPSAM

The purpose of the Single Intact Stability Index (SISI) is to quantitatively evaluate the ten IMO intact stability parameters of passenger ships. As shown in Figure 3, the assessment standards of SISI are proposed according to the value of SISI. In the SISI standards, the stability of passenger ships is classified into five levels. The details of the SISI standard are shown in Table 7. A level of “Severe Risk”, is when more than 50% of stability parameters are not in compliance with the IMP requirements. Passenger ships’ crew are advised to take prompt action to improve ships’ stability with a level of “Severe Risk”. For the level of “Danger”, less than 50% of the ten IMO stability parameters do not satisfy the IMO stability regulations, thus suitable actions to increase the stability index should be carried out. In the other three levels, the SISI is over 1.0 meaning that all IMO stability parameters satisfy the requirements for stability safety. In the level of “Minimum Safety Condition”, although the SISI is over 1.0, it is hard for passenger ships to ensure stability safety while navigating on the sea. Thus, ships’ crew should carefully monitor the SISI and improve the stability conditions to fall in the range of SISI 1.0 to 1.2.

Table 8 shows the results of the SISI assessment process as Phase Ⅲ in the module of IPSAM using the parameters of Table 6. As shown in the results of Table 6, the SISI should be less than 1.0 in loading conditions Nos. 1, 2, and 3 because these do not comply with IMO intact stability regulations. In these loading conditions, the stability parameter indexes for the IMO intact stability parameters not complying with requirements indicate less than 1.0, as shown in Table 8. As shown in Table 6, all stability parameters satisfy the IMO stability regulations for the first time with loading case No.4. Thus, the IMO intact stability parameters of the loading case were used as $y_{i-1\;\left(SafetyLimit\right)}$, then their indexes are denoted 1.0 as shown in Table 8. Accordingly, the Single Intact Stability Index (SISI) successfully indicated the level of stability safety as under 1.0 in the three noncompliant loading conditions No. 1, 2, and 3 as 0.44, 0.50, and 0.67 respectively. Hence, IPSAM was a convenient and easy decision support system for passenger ships’ crew to alert the risk of the ships’ stability risk in the condition of ships not satisfying a stability parameter indicating a SISI under 1.0.

However, there are limitations in the process of leveling the stability conditions between “Severe Risk” and “Danger” according to SISI which is determined as the average of the stability indexes. In loading case No. 2 in Table 8, six IMO intact stability parameters are not in compliance with their requirements among the total of nine parameters. This means that less than 50% of IMO intact stability parameters comply with their requirements. Thus, the SISI of loading case No. 2 should be under 0.5 to indicate the level of stability safety as “Server Risk” as shown in Table 7. The average of each stability index could not exactly reflect the number of IMO stability parameters that are not in compliance with the SISI standard because of the high indexes compensating the low indexes in the calculation of their average. In future studies, it is necessary to find all loading cases that only satisfy 50% of the IMO intact stability parameters to define exactly the standard parameters for indexing the role of $y_{i-1\;\left(SafetyLimit\right)}$ in defining all parameters that satisfy their requirements. 

## 5. Conclusions

IPSAM is developed in the present study to easily evaluate passenger ships’ IMO intact stability parameters according to GM and propose the present stability safety status as SISI. This module supports the decision of passenger ships’ crew to take actions to improve ships’ stability as an effective auxiliary method. Based on ten IMO intact stability parameters of 331 loading conditions of 11 passenger ships, empirical formulas were derived. Finally, 20 loading conditions of the passenger ship were evaluated by using IPSAM to verify them. The following conclusions and recommendations for future studies can be drawn.

- The IPSAM composed the three phases (intact stability parameters calculation process, indexing process, SISI assessment process) for evaluating IMO intact stability parameters as shown in Figure 3. The introduction of IPSAM for types of passenger ships such as car carriers and Ropax is considered as an effective auxiliary method for crews who have limited knowledge for calculating and evaluating IMO stability parameters.
- The derived empirical formulas except for $Angle_{maxGZ}$ according to the variable of GM of IPSAM successfully calculate stability parameters to distinguish whether they are compliant with the IMO intact stability requirements or not in 20 passenger ships’ loading conditions as shown in Table 5. The average error ratio of all IMO intact stability parameters calculated by the eight empirical formulas is 17.9%.
- There is a limitation of the derivation of the simple formula to reliably calculate the angle of heel at the maximum point called $Angle_{maxGZ}$ of the sinusoidal function of the GZ curve according to a single variable of GM calculated by the roll-period measured by a sensor in real-time herein. To overcome the limitation of the empirical formula of $Angle_{maxGZ}$, additional further studies with the consideration of additional ships’ variables while operating them on the sea are required in the future.
- The SISI provides passenger ship operators with the level of ships’ stability safety as five categories. The SISI can evaluate the safety of ship stability more efficiently. As shown in Table 8, SISI accurately indicates unsatisfied loading conditions with IMO requirements as loading conditions Nos. 1, 2, and 3 as under 1.0.
- The average of stability indexes has a limitation in the process of determining the level of stability between “Severe Risk” and “Danger” because of high indexes compensating low indexes in the calculation of their average. The SISI of loading case NO. 2 which had less than 50% of IMO intact stability parameters complying with their requirements should be under 0.5 in Table 8.
- In future studies, to improve the reliability of empirical formulas of IPSAM, additional loading conditions of various types of passenger ships will be included. Additionally, IPSAM using GM calculated by using a sensor measuring the roll period of a passenger ship will be carried out to monitor the level of passenger ships’ stability safety in real-time.

## Figures and Tables

**Table 1:** International Maritime Organization (IMO) intact stability parameters and criteria.

No	Stability Parameter	Criteria
1	GM	0.150 m
2	$GZ_{30deg}$	0.200 m
3	$Angle_{maxGZ}$	25 deg
4	$Area_{0-30deg}$	0.055 m·rad
5	$Area_{0-40deg}$	0.090 m·rad
6	$Area_{30-40deg}$	0.030 m·rad
7	$Area_{ratio}$	1.0
8	$\varphi_{0}$	16° and 80% of angle of deck edge immersion, whichever is less
9	$Angle_{passenger}$	10 deg
10	$Angle_{turning}$	10 deg

**Table 2:** Particulars of the model ships.

No	Length B.P. (m)	Breadth (m)	Depth (m)	Design Draught (m)	Full Displacement (ton)	Block Coefficient
1	151.2	26.4	10.8	5.5	13,452.1	0.712
2	149.6	24.3	11.5	5.8	12,727.3	0.673
3	170.0	26.5	13.0	6.2	18,032.5	0.754
4	192.0	34.0	15.2	7.6	23,665.8	0.765
5	171.0	27.0	13.2	6.8	16,044.7	0.722
6	138.5	24.1	10.5	6.3	11,135.0	0.710
7	132.0	22.0	11.1	6.4	9907.5	0.695
8	104.0	17.8	9.5	5.2	6434.6	0.501
9	120.0	19.4	9.8	4.8	9122.2	0.675
10	94.0	15.6	8.5	4.3	4626.7	0.475
11	93.0	14.5	8.2	4.1	4318.8	0.486

**Table 3:** Empirical formulas according to the GM.

Stability Parameter	Empirical Formula According to GM	${R^{2}}^{\;}$
$GZ_{30deg}$	$y_{1}=-0.0854x^{2}{}_{GM}+0.7299x$	0.7657
$Angle_{maxGZ}$	$y_{2}=-1.0021x^{2}{}_{GM}-0.2328x+48.32$	0.7419
$Area_{0-30deg}$	$y_{3}=0.1181x_{GM}+0.0291$	0.9525
$Area_{0-40deg}$	$y_{4}=-0.007x^{3}{}_{GM}+0.0197x^{2}{}_{GM}+0.2265x$	0.9112
$Area_{30-40deg}$	$y_{5}=-0.0166x^{2}{}_{GM}+0.1302x$	0.8126
$Area_{ratio}$	$y_{6}=-0.4817x_{GM}+4.6225$	0.0993
Area c	$y_{7}==0.0108x^{2}{}_{GM}+0.0446x$	0.8754
Area d	$y_{8}=-0.0228x^{2}{}_{GM}+0.2968x$	0.7707
$\varphi_{0}$	$y_{9}=8.0468x_{GM}^{-1.008}$	0.8192
$Angle_{passenger}$	$y_{10}=2.3876x_{GM}^{-0.357}$	0.8997
$Angle_{turning}$	$y_{11}=-4.775\ln\left(x\right)+8.0008$	0.9271

**Table 4:** Particulars of the passenger ship.

Items	Car Ferry	
Length O. A.	189.0 m	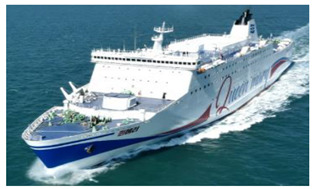
Length B. P.	171.0 m	
Breadth (B)	15.2 m	
Draft (d)	6.7 m	
Full Displacement	16,044.7 ton	
$C_{b}<$	0.511	

**Table 5:** IMO intact stability parameters using the principal methods.

No	Compliance wih IMO Reg	GM (m) [0.15]	*GZ*_30*deg*_ (m) [0.20]	*Area*_0–30_ (m·rad) [0.055]	*Area*_0–40_ (m·rad) [0.090]	*Area*_30–40_ (m·rad) [0.030]	φ__0_ (deg)	*Area_ratio_* (d/c) [1.0]	*Angle_passenger_* (*deg*)	*Angle* *_turning_* (*deg*)
1	x	0.100	0.056	0.032	0.019	0.011	42.406	1.942	4.407	11.522
2	x	0.200	0.118	0.044	0.037	0.024	23.561	2.257	3.931	10.803
3	x	0.300	0.202	0.056	0.061	0.036	14.885	2.911	3.160	10.458
4	o	0.400	0.220	0.068	0.093	0.039	11.450	3.138	2.889	8.361
5	o	0.500	0.276	0.081	0.110	0.057	8.750	3.727	2.518	8.198
6	o	0.600	0.320	0.080	0.121	0.057	7.314	3.845	2.507	8.073
7	o	0.700	0.345	0.088	0.142	0.074	5.668	4.606	2.478	7.486
8	o	0.781	0.465	0.108	0.172	0.074	5.342	4.375	1.984	7.259
9	o	0.800	0.435	0.120	0.186	0.070	5.070	4.488	2.461	6.229
10	o	0.900	0.463	0.110	0.190	0.085	4.092	4.866	2.155	6.479
11	o	1.000	0.556	0.138	0.230	0.099	3.782	5.431	1.876	6.461
12	o	1.100	0.602	0.141	0.246	0.122	4.365	5.357	2.058	6.563
13	o	1.200	0.630	0.147	0.265	0.107	3.500	5.690	1.927	6.636
14	o	1.300	0.606	0.154	0.284	0.133	3.374	5.588	1.664	6.203
15	o	1.381	0.811	0.158	0.298	0.141	2.745	5.869	1.693	5.791
16	o	1.382	0.646	0.153	0.293	0.128	3.242	5.537	1.676	6.090
17	o	1.418	0.718	0.152	0.296	0.127	2.769	5.271	1.750	6.290
18	o	1.887	0.922	0.201	0.399	0.168	2.345	6.783	1.670	5.733
19	o	1.884	0.816	0.196	0.394	0.167	2.055	5.471	1.469	5.947
20	o	1.905	0.899	0.193	0.393	0.159	2.286	5.481	1.546	6.124

: Incompliant with IMO Stability Regulations. o: Compliant with IMO Stability Regulations, x: Incompliant with IMO Stability Regulations.

**Table 6:** IMO stability parameters using the empirical formulas and error ratio.

No	GM (m) [0.15]	*GZ*_30 *deg*_ (m) [0.20]	Error Ratio (%)	*Area*_0–30_ (m·rad) [0.055]	Error Ratio (%)	*Area*_0–40_ (m·rad) [0.090]	Error Ratio (%)	*Area*_30–40_ (m·rad) [0.030]	Error Ratio (%)	φ__0_ (*deg*)	Error Ratio (%)	*Area_ratio_* (d/c) [1.0]	Error Ratio (%)	*Angle_passenger_* (*deg*)	Error Ratio (%)	*Angle_turning_* (*deg*)	Error Ratio (%)
1	0.100	0.072	28.8	0.041	27.0	0.023	19.3	0.013	18.4	51.406	21.2	2.172	11.8	5.432	23.3	12.776	10.9
2	0.200	0.143	21.1	0.053	20.3	0.046	23.9	0.025	7.6	25.561	8.5	2.797	23.9	4.241	7.9	11.338	4.9
3	0.300	0.211	4.4	0.065	16.2	0.070	14.9	0.038	3.3	16.985	14.1	3.371	15.8	3.670	16.1	10.498	0.4
4	0.400	0.278	26.3	0.076	11.7	0.093	0.1	0.049	25.4	12.710	11.0	3.888	23.9	3.312	14.6	9.901	18.4
5	0.500	0.344	24.4	0.088	9.2	0.117	6.7	0.061	7.0	10.150	16.0	4.339	16.4	3.058	21.5	9.438	15.1
6	0.600	0.407	27.2	0.100	25.0	0.141	16.5	0.072	26.3	8.446	15.5	4.738	23.2	2.865	14.3	9.060	12.2
7	0.700	0.469	35.9	0.112	27.3	0.166	16.9	0.083	12.0	7.230	27.6	5.081	10.3	2.712	9.4	8.740	16.8
8	0.781	0.518	11.5	0.121	12.4	0.186	7.8	0.092	24.5	6.474	21.2	5.327	21.8	2.608	31.5	8.513	17.3
9	0.800	0.529	21.6	0.124	3.4	0.190	2.2	0.094	33.4	6.320	24.7	5.384	19.9	2.586	5.1	8.464	35.9
10	0.900	0.588	27.0	0.135	22.7	0.215	13.9	0.104	22.1	5.612	37.1	5.651	16.1	2.479	15.1	8.219	26.8
11	1.000	0.645	16.0	0.147	6.4	0.239	3.9	0.114	14.3	5.047	33.5	5.891	8.5	2.388	27.3	8.001	23.8
12	1.100	0.700	16.3	0.159	12.8	0.264	7.3	0.123	1.2	4.585	5.0	6.107	14.0	2.308	12.2	7.803	18.9
13	1.200	0.753	19.6	0.171	15.9	0.288	8.8	0.132	24.2	4.200	20.0	6.302	10.8	2.237	16.1	7.623	14.9
14	1.300	0.805	32.8	0.183	18.7	0.312	10.2	0.141	6.0	3.874	14.8	6.481	15.9	2.174	30.7	7.457	20.2
15	1.381	0.845	4.2	0.192	21.7	0.332	11.5	0.148	5.3	3.645	32.8	6.709	14.3	2.128	25.7	7.331	26.6
16	1.382	0.846	30.9	0.192	25.9	0.332	13.5	0.148	15.6	3.642	12.3	6.489	17.2	2.127	26.9	7.330	20.4
17	1.418	0.863	20.2	0.197	29.7	0.341	15.2	0.151	18.9	3.549	28.2	6.794	28.9	2.108	20.5	7.277	15.7
18	1.887	1.072	16.3	0.251	25.1	0.449	12.6	0.188	11.2	2.664	13.6	8.017	18.1	1.904	14.0	6.684	16.6
19	1.884	1.072	31.3	0.252	28.5	0.450	14.2	0.186	11.3	2.665	29.7	7.321	33.8	1.904	29.6	6.687	12.4
20	1.905	1.081	20.2	0.254	31.7	0.455	15.6	0.188	18.3	2.636	15.3	6.931	26.5	1.897	22.7	6.664	8.8

: Incompliant with IMO Stability Regulation,  : Error Ratio over 20%,  : Error Ratio over 30%.

**Table 7:** Details of the standards of the Single Intact Stability Index (SISI).

Level of Stability Safety	SISI	Compliance with Ten IMO Intact Stability Requirements
**Severe Risk**	**0.0–0.5**	Less than 50% of the nine IMO intact stability parameters comply with their requirements for stability safety However, SISI could indicate “Severe Risk” when low indexes compensate high indexes. This is the limitation of the averaging method for the stability indexes
**Danger**	**0.5–1.0**	More than 50% of the nine IMO intact stability parameters comply with the requirements for stability safety However, SISI could indicate “Danger” when high indexes compensate low indexes. This is the limitation of the averaging method for the stability indexes
**Minimum Safety Condition**	**1.0–1.2**	All parameters satisfy the IMO stability parameter requirements
**Normal Safety Condition**	**1.2–2.0**	
**Considerable Safety**	**Over 2.0**	

**Table 8:** IMO intact stability parameter indexes SP_i_ and SISI.

No	IMO Intact Stability Parameter Indexes SP_i_										SISI
	Compliance wih IMO Reg	GM	*GZ*_30 *deg*_	*Area*_0–30_	*Area*_0–40_	*Area*_30–40_	φ__0_	*Area*_ratio_ (d/c)	*Angle* *_passenger_*	*Angle* *_turning_*	
1	x	0.33	0.18	0.37	0.13	0.22	0.76	0.70	0.84	0.43	0.44
2	x	0.58	0.36	0.48	0.26	0.42	0.16	0.81	0.93	0.47	0.50
3	x	0.74	0.57	0.74	0.39	0.71	0.46	0.91	0.97	0.49	0.67
4	o	1.00	1.00	1.00	1.00	1.00	1.00	1.00	1.00	1.00	1.00
5	o	1.03	1.06	1.05	1.05	1.06	1.31	1.11	1.11	1.09	1.10
6	o	1.10	1.12	1.09	1.09	1.11	1.52	1.20	1.19	1.17	1.18
7	o	1.17	1.18	1.14	1.14	1.16	1.67	1.29	1.26	1.23	1.25
8	o	1.22	1.22	1.17	1.18	1.20	1.76	1.34	1.30	1.28	1.30
9	o	1.23	1.23	1.18	1.19	1.21	1.78	1.36	1.31	1.29	1.31
10	o	1.30	1.29	1.22	1.24	1.25	1.86	1.42	1.36	1.34	1.37
11	o	1.37	1.34	1.27	1.28	1.30	1.93	1.48	1.40	1.38	1.42
12	o	1.44	1.39	1.31	1.33	1.34	1.99	1.53	1.43	1.42	1.47
13	o	1.51	1.44	1.36	1.38	1.38	2.07	1.58	1.46	1.46	1.51
14	o	1.58	1.49	1.40	1.43	1.42	2.14	1.62	1.49	1.49	1.56
15	o	1.63	1.53	1.44	1.46	1.46	2.19	1.68	1.51	1.51	1.60
16	o	1.63	1.53	1.44	1.46	1.46	2.19	1.62	1.51	1.51	1.60
17	o	1.66	1.55	1.45	1.48	1.47	2.21	1.70	1.52	1.52	1.62
18	o	1.98	1.74	1.66	1.69	1.64	2.41	1.99	1.61	1.64	1.82
19	o	1.98	1.74	1.66	1.69	1.63	2.41	1.82	1.61	1.64	1.80
20	o	1.99	1.75	1.67	1.70	1.64	2.41	1.73	1.61	1.65	1.79

: Incompliant with IMO Stability Regulation. o: Compliant with IMO Stability Regulation, x: Incompliant with IMO Stability Regulation.
